# Masticatory performance in patients undergoing free fibula flap for mandible reconstruction

**DOI:** 10.1186/s12903-022-02114-4

**Published:** 2022-03-18

**Authors:** Jun Zhang, Yujing Wang, Lulu Yuan, Weiren Wang

**Affiliations:** Department of Oromaxillofacial-Head and Neck Surgery and Department of Oral and Maxillofacial Surgery, School and Hospital of Stomatology, Liaoning Provincial Key Laboratory of Oral Diseases, China Medical University, Shenyang, China

**Keywords:** Masticatory performance, Mandible reconstruction, Oral squamous cell carcinoma, Free fibula flap, Segmental resection

## Abstract

**Background:**

To explore the masticatory performance in patients undergoing an osteo(cutaneous) free fibula (OFF) flap for mandible reconstruction by a prospective design.

**Methods:**

A total of 56 patients who had undergone OFF flap reconstructions for mandibular reconstruction secondary to malignant (squamous cell carcinoma) or benign (ameloblastoma) tumor resection were prospectively enrolled. They were asked to complete the masticatory performance test by the weigh method and the chew domain of the University of Washington quality of life questionnaire (version 4) preoperatively and at 3, 6, and 12 months postoperatively. The pair nonparametric test was used to analyze the dynamic change of masticatory performance and subjective chew function.

**Results:**

Fifty-one patients were included for analysis finally. The mean masticatory performance for patients with malignant tumors were 53.4% ± 10.3%, 36.4% ± 10.3%, 42.6% ± 9.6%, 52.8% ± 10.9%, and 53.1% ± 11.8% preoperatively, at 2 weeks, 3 months, 6 months, and 12 months postoperatively, respectively. Compared with the preoperative level, the masticatory performance had a significant reduction immediately after surgery (*p* < 0.001), followed by a return to the baseline level within three months. A similar trend was noted for those with benign tumors. The mean score of chew domain for patients with malignant tumors were 100 ± 0, 54.3 ± 32.9, 81.4 ± 24.5, and 92.9 ± 17.8 preoperatively, at 3 months, 6 months, and 12 months postoperatively, respectively. Compared with the preoperative level, the subjective chew function was greatly affected within the first three months (*p* < 0.001), and it gradually recovered to the baseline level in the following nine months. A similar trend was noted in patients with benign tumors.

**Conclusions:**

The masticatory performance and subjective chew function was significantly affected after OFF flap reconstructions in the short term, but both recovered to the preoperative levels within 9–12 months.

## Introduction

The mandible is key for normal oral function, but segmental resection of the mandible, which is occasionally required owing to tumor invasion, trauma or others reasons, may significantly hamper it [1]. Since it was firstly introduced by Taylor et al.[2], the osteo(cutaneous) free fibula (OFF) flap is the preferred method for mandible reconstruction currently [3]. The OFF flap provides a reliable skin paddle for covering soft tissue defects, and more importantly, it offers an adequate length of thick cortical bone allowing dental implant placement [4]. The main goal of mandible reconstruction is to achieve good objective and subjective oral function.

Several authors have evaluated the quality of life (QoL) and functional results in patients undergoing OFF flaps for mandible reconstruction [5–10]. Ni et al. [5] found all the nine fields of the SF-36 questionnaire, including physical functioning, role-physical, bodily pain, general health, vitality, social functioning, role-emotional, mental health, and reported health transition, had acceptable scores. Similar results are also reported by others [6, 7, 10].

Masticatory performance is an important aspect of assessing the oral function [11]. To the best of our knowledge, few studies have analyzed the masticatory performance following OFF flap reconstruction [8, 9]. Ciocca et al. [8] enrolled 10 patients of whom 5 received removable prostheses and 5 received implant-supported fixed prostheses; the fixed prostheses group had significantly better masticatory performance than the removable prostheses group. In a study by Kumar et al. [9] including 10 participants who had undergone implant placement, the authors observed that there were no significant differences in the masticatory performance between the rehabilitated side and the normal side in both subjective and electronic assessments. Moreover, not all patients with OFF flap reconstructions proceed for prosthetic rehabilitation; the masticatory performance of these patients also need attention. More importantly, masticatory performance was objective and was closely related to daily life, it was significantly associated with subjective chewing ability. Then it was necessary to take an approach from both subjective and objective evaluation, and there was a hypothesis that OFF reconstruction wound recover masticatory function after surgery equally to that before surgery.

Therefore, in this prospective study, we aimed to explore the improvement of masticatory performance and the quality of life over time in patients who underwent OFF flaps for mandible reconstructions.

## Patients and methods

### Ethical consideration

The Affiliated Stomatology Hospital Hospital of China Medical University institutional research committee approved this study, and all participants provided written informed consent. All methods were performed in accordance with the relevant guidelines and regulations and all procedures on patients were in accordance with the ethical standards of the institutional and/or national research committee and the 1964 Helsinki Declaration and its later amendments or comparable ethical standards.

### Patient selection

From January 2018 to August 2020, a total of 56 consecutive patients underwent OFF flaps for mandible reconstructions at our hospital (Table 1). Of these, 38 underwent mandibular resection owing to oral cancer and 18, owing to ameloblastoma. After receiving clear explanations of our requirements, all the patients agreed to participate in this study. Demographic and pathologic information of these patients was collected and analyzed.

**Table 1:** Patients’ basic information at the base line and end point

Variable	Base line (n = 56)	End point (n = 51)
Age	49.2 (31–65)	49.6 (31–65)
Sex		
Male	41	39
Female	15	12
Disease		
Oral cancer	38	35
Ameloblastoma	18	16
Mandible defect type		
H	14	14
L	25	25
LC	11	10
C	6	2

The patients were asked to complete the masticatory performance test and the chew domain of the University of Washington quality of life (UW-QoL) questionnaire (version 4) preoperatively, and at 3 months, 6 months, and 12 months postoperatively.

In the course of our research, 5 patients were excluded owing to incomplete objective or subjective evaluations. Finally, 39 male and 12 female participants were included. The mean age was 49.6 (range 31–65) years. The median number of remaining teeth before and after treatment was 24 (range 18–30) and 16 (range 10–24), respectively.

Thirty-five patients had been diagnosed with primary oral cancer. Squamous cell carcinoma of the mandibular gingiva was diagnosed in 28 cases, while that of the floor of the mouth was diagnosed in 7. None of these patients had received any treatment previously. They underwent primary tumor resection, neck dissection, and OFF flap reconstruction simultaneously.

Sixteen patients had been diagnosed with ameloblastoma. They underwent primary tumor resection and OFF flap reconstruction simultaneously.

### Mandible defect classification

The mandibular defect was classified according to the HCL method [12]; where, H referred to any defect that was located unilaterally and included the condyle, L referred to any defect that was located unilaterally and excluded the condyle, and C referred to central defects between the bilateral canines.

In patients with malignant disease, mandible defect types were assessed as H in 8 patients, L in 15, LC in 10, and C in 2. Postoperative radiotherapy was also performed for all these patients. No patient received removable or fixed denture rehabilitation owing to the possibility of disease recurrence within 2 years after surgery or poor economic status.

In patients with benign disease, mandible defect types were assessed to be H in 6 patients and L in 10. Eight patients underwent removable denture rehabilitation 6 months postoperatively.

### Masticatory performance test

There was no consensus on the optimal evaluation method. Based on official instruction [13], during each test, the patients were required to chew 5.0 g of peanuts for 20 s, then, following a thorough rinse, they were asked to spit in a measuring cylinder. The chewed food was sieved through a mesh with 2 mm perforations. The residue in the mesh was weighed. The masticatory performance was defined as the percentage of the difference between 5.0 g and the weight of the residue.

### Subjective mastication assessment

The UW-QoL questionnaire is one of the most reliable questionnaires used for evaluating the QoL [14, 15]. Chew domain (Table 2) is one of the twelve specific head-and-neck domains. The response could be one of three choices, which are scored from 0 (worst) to 100 (best). The higher the score, the better is the QoL.

**Table 2:** Detailed information of the chewing domain of the University of Washington quality of life (UW-QoL) questionnaire (version 4)

Chewing domain	Score
I can chew as well as ever	100
I can eat soft solids but can not chew some foods	50
I can not even chew soft solids	0

### Statistical analysis

The Kolmogorov–Smirnov test was used for normality analysis. The pair nonparametric Wilcoxon test adapted with Bonferroni correction was used to analyze the dynamic change of masticatory performance and subjective chew function. Both masticatory performance and subjective chew function were compared in patients with different clinicopathologic characteristics using Mann–Whitney U test. All statistical analyses were performed using Statistical Package for the Social Sciences (SPSS) version 20.0, and the level of statistical significance was set at *p* < 0.05.

## Results

### Masticatory performance in patients with malignant tumors

**Fig. 1:**
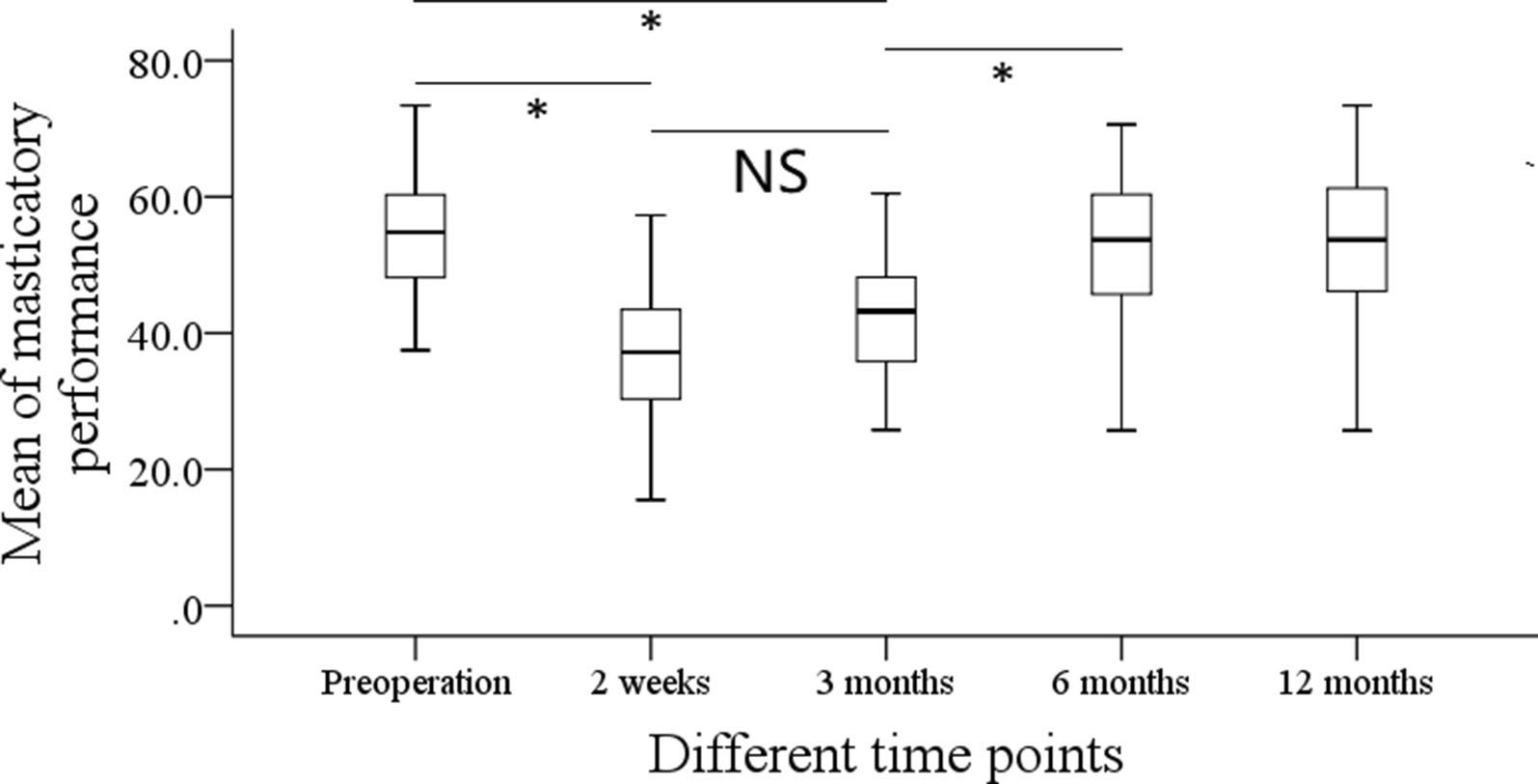
Masticatory performance in patients with malignant tumors at different time points

Before operation, the mean masticatory performance was 53.4% ± 10.3%, and after the operation, the masticatory performance were 36.4% ± 10.3%, 42.6% ± 9.6%, 52.8% ± 10.9%, and 53.1% ± 11.8% at 2 weeks, 3 months, 6 months, and 12 months, respectively. Compared with the preoperative level, the masticatory performance had a significant reduction immediately after surgery (*p* < 0.001), it maintained the status quo till the third month, then it began to recover, the masticatory performance at the sixth month was comparable with that at the baseline level (*p* > 0.05) (Fig. 1).

### Masticatory performance in patients with benign tumors

**Fig. 2:**
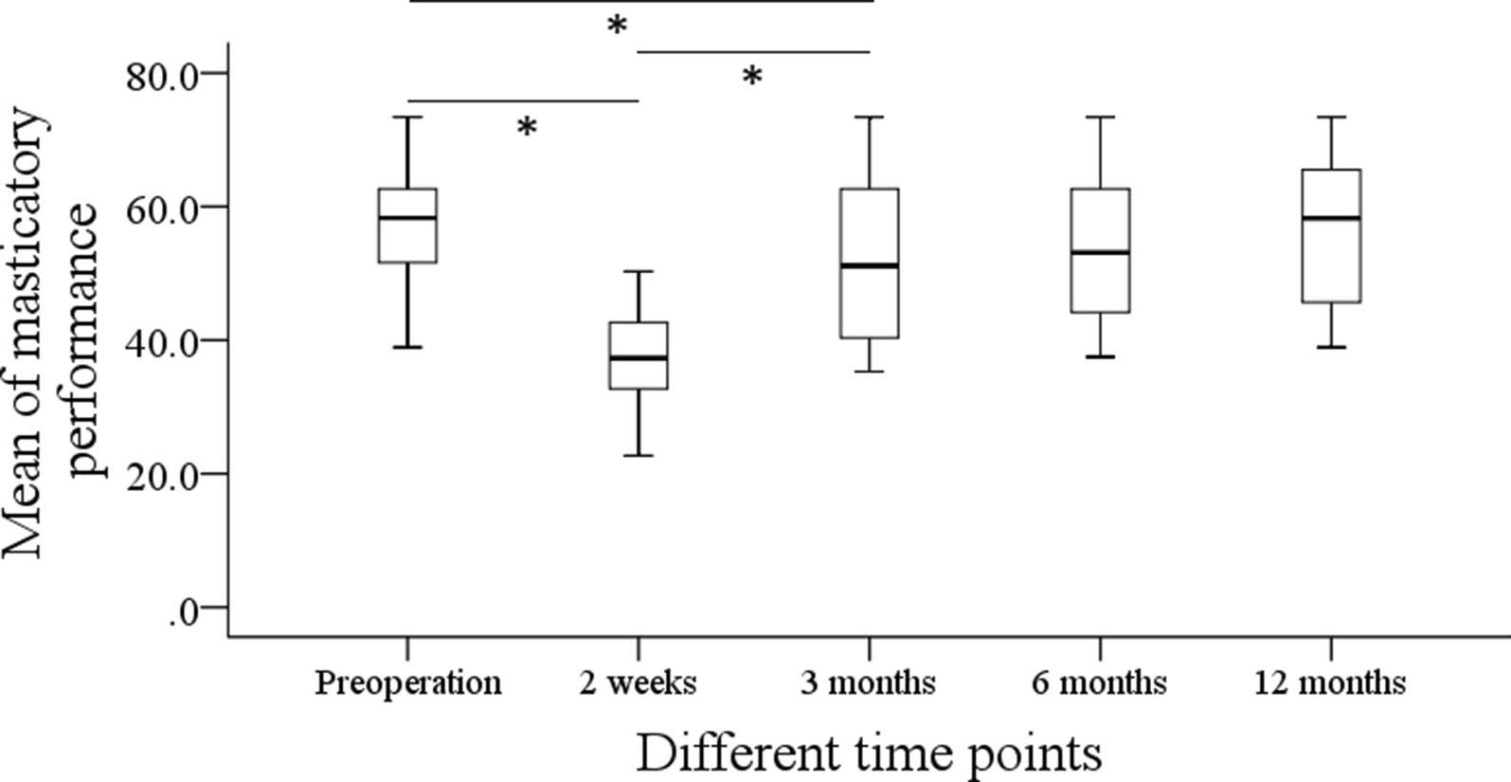
Masticatory performance in patients with benign tumors at different time points

Before the operation, the mean masticatory performance was 56.5% ± 10.3%. After the operation, the mean masticatory performance were 37.3% ± 7.7%, 51.9% ± 12.1%, 54.1% ± 11.4%, and 56.5% ± 11.8% at 2 weeks, 3 months, 6 months, and 12 months, respectively. Compared with the preoperative level, the masticatory performance had a significant reduction immediately after surgery (*p* < 0.001), but it began to recover from then on, the masticatory performance at the third month was significantly better than that at the 2nd week (*p* < 0.001) but lower than that at the sixth month (*p* < 0.001) which was comparable with that at the baseline level (Fig. 2).

The patients were divided into two groups based on different clinicopathologic characteristics, and the masticatory performance between the two groups did not differ significantly at all time points (all *p* > 0.05, Table 3).

**Table 3:** Comparison of masticatory performance in patients with different clinicopathologic characteristics

	Preoperation	Postoperative		
		3 months	6 months	12 months
Denture rehabilitation	56.7 ± 9.5	52.3 ± 13.4	54.1 ± 11.9	54.9 ± 10.7
No denture rehabilitation	56.3 ± 11.7	51.4 ± 11.6	54.0 ± 11.6	58.1 ± 13.3
*p*	0.936	0.877	0.993	0.595
Benign tumor	56.0 ± 10.3	52.4 ± 14.3	55.2 ± 10.5	56.3 ± 10.6
Malignant tumor	56.5 ± 10.9	51.1 ± 10.7	53.5 ± 13.0	58.2 ± 13.4
p	0.901	0.845	0.884	0.637
H + L^a^	56.3 ± 9.8	53.2 ± 10.3	54.6 ± 11.0	58.5 ± 10.9
LC + C	56.6 ± 11.4	46.2 ± 14.7	52.1 ± 12.5	54.5 ± 13.1
*p*	0.944	0.123	0.667	0.700
Male	56.5 ± 10.0	51.8 ± 12.1	53.8 ± 11.7	57.4 ± 12.6
Female	55.9 ± 11.2	50.7 ± 12.9	54.7 ± 11.8	58.2 ± 11.4
*p*	0.876	0.776	0.825	0.884
Number of teeth ≤ median	55.9 ± 11.1	51.1 ± 11.8	52.2 ± 10.6	56.7 ± 11.2
Number of teeth > median	56.7 ± 10.3	51.9 ± 13.2	55.8 ± 12.9	58.5 ± 12.8
*p*	0.687	0.727	0.356	0.572

^a^H/L/LC/C: Mandible defect type

### Subjective mastication assessment in patients with malignant tumors

**Fig. 3:**
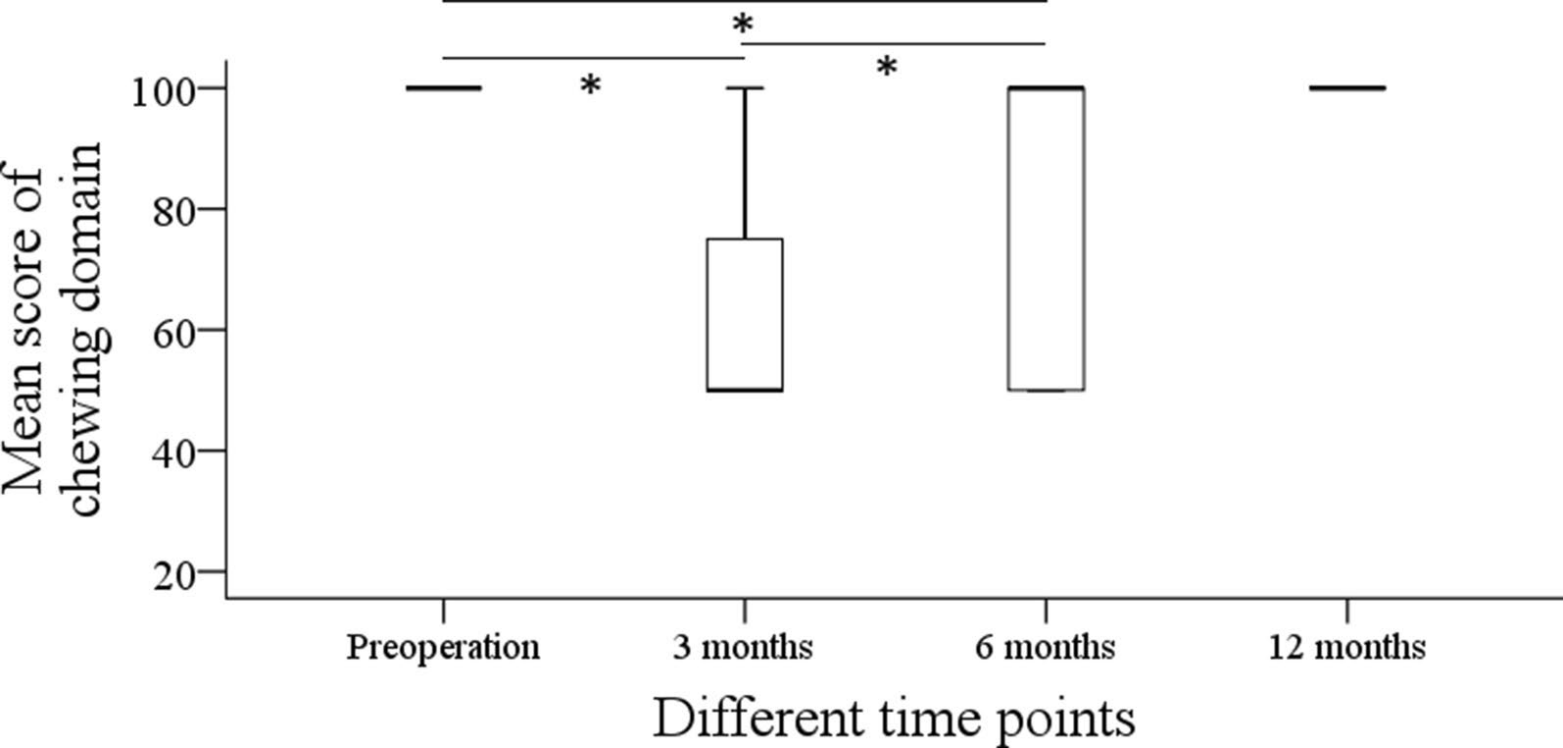
Subjective mastication assessment in patients with malignant tumors at different time points

Before operation, the mean score of the chew domain was 100 ± 0. After the operation, the mean scores of chew domain were 54.3 ± 32.9, 81.4 ± 24.5, and 92.9 ± 17.8 at 3 months, 6 months, and 12 months, respectively. Compared with the preoperative level, the subjective chew function was greatly affected in the first three months (*p* < 0.001), and it gradually recovered to the baseline level in the following nine months (Fig. 3).

### Subjective mastication assessment in benign patients

**Fig. 4:**
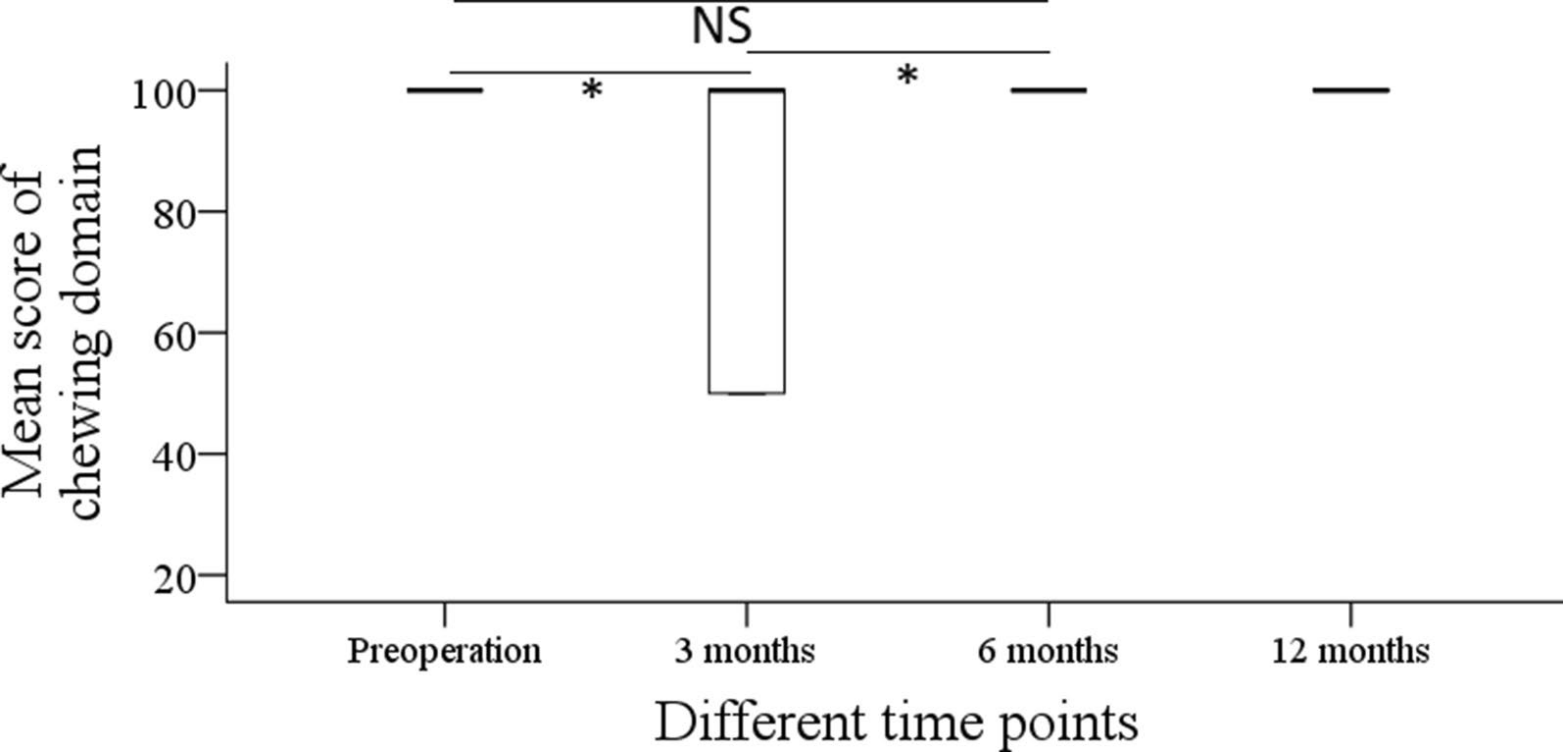
Subjective mastication assessment in patients with benign tumors at different time points

Before operation, the mean score of the chew domain was 100 ± 0. After the operation, the mean scores of chew domain were 78.1 ± 25.6, 93.8 ± 17.1, and 100 ± 0 at 3 months, 6 months, and 12 months, respectively. Compared with the preoperative level, the subjective chew function was greatly affected in the first three months (*p* < 0.001), and it gradually recovered to the baseline level within six months (Fig. 4).

The patients were divided into two groups based on different clinicopathologic characteristics, and the subjective chew function between the two groups did not differ significantly at all time points (all *p* > 0.05, Table 4).

**Table 4:** Comparison of subjective chew function in patients with different clinicopathologic characteristics

	Preoperation	Postoperative		
		3 months	6 months	12 months
Denture rehabilitation	100 ± 0	81.3 ± 25.9	93.8 ± 17.7	100 ± 0
No denture rehabilitation	100 ± 0	75.0 ± 26.7	93.8 ± 17.7	100 ± 0
*p*	1.000	0.642	1.000	1.000
Benign tumor	100 ± 0	82.6 ± 24.6	94.5 ± 15.6	100 ± 0
Malignant tumor	100 ± 0	72.9 ± 28.0	93.5 ± 19.8	100 ± 0
*p*	1.000	0.110	0.874	1.000
H + L*	100 ± 0	80.3 ± 17.8	94.2 ± 18.3	100 ± 0
LC + C	100 ± 0	61.9 ± 34.8	92.5 ± 17.1	100 ± 0
*p*	1.000	0.057	0.675	1.000
Male	100 ± 0	77.4 ± 23.1	94.5 ± 19.4	100 ± 0
Female	100 ± 0	73.4 ± 29.5	91.5 ± 16.0	100 ± 0
*p*	1.000	0.773	0.888	1.000
Number of teeth ≤ median	100 ± 0	74.6 ± 25.2	89.8 ± 21.1	100 ± 0
Number of teeth > median	100 ± 0	78.2 ± 27.4	97.8 ± 14.3	100 ± 0
*p*	1.000	0.888	0.513	1.000

## Discussion

This study dynamically analyzed masticatory performance and subjective chew function in patients who underwent OFF flaps for mandible reconstruction. The most important finding in current study was that the masticatory performance and subjective chew function were significantly affected after the OFF flap reconstructions in short term, but both parameters recovered to the preoperative level within 9–12 months. It emphasized the reliability and practice of OFF flaps in mandible reconstruction.

Masticatory performance refers to the ability of grinding food within a specific time. While several methods are available for assessing the masticatory performance [8, 9, 16], and the weighing method is the most frequently used one [13]. Ciocca et al. [8], the first to evaluate masticatory performance in patients undergoing OFF flaps reconstruction, enrolled 20 patients of whom 5 received removable dentures, 5 received fixed dentures, and the remaining 10 acted as controls. They found that the controls had a masticatory performance of 91.4% ± 8.7%, which was significantly higher than that in the fixed and removable prostheses groups (67.4% ± 28.9%, 28.0% ± 28.5%, respectively). They suggested that rehabilitation with fixed dentures was superior to that with removable dentures. However, the study had limitations, such as there was no comparison of preoperative masticatory performance, the lack of information on how many patients underwent radiotherapy, and the duration between surgery and denture rehabilitation remained unknown; all of these could have affected the results.

Kumar et al. [9] found that the subjective and objective masticatory performance in normal side and rehabilitated sides, compared using gum wafers, were similar. However, in addition to the limited sample size, this study did not enroll patients with fixed dentures or those without dentures, and it did not distinguish between patients with malignant and benign tumors.

Recently, de Groot et al. [18] analyzed the functional results in six patients who underwent digitally planned OFF flap reconstructions. The authors reported that, compared with traditional methods, digitally planned reconstructions provide better mixing ability but similar maximum mouth opening and bite force. The finding was interesting, but this study enrolled patients with both maxillary and mandibular reconstructions.

All these reports assessed the masticatory performance at a certain time point only. As can be predicted, the masticatory performance was influenced by oral pain, local inflammation, and so on [18]. Then it was believed that there would be a dynamic change of masticatory performance after surgery, but unfortunately this issue was rarely evaluated. de Groot et al. [19] might be the first to determine the masticatory performance in various phases, in this prospective study, 123 patients were included, masticatory performance was drastically affected by oral cancer and its treatment, but it could recover to pretreatment levels in patients who survive for 5 years. A higher number of occlusal units, having full dentures or better, elevated maximum bite force, increased maximum mouth opening, and having a maxillary rather than mandibular provided aid in improving the masticatory performance. We noted masticatory performance decreased shortly after surgery, but slowly increased to preoperative level independent of the nature of the pathology. This seems to suggest that OFF flap reconstructions can preserve the chew function to the most extent, and that this effect has no relation with the type of denture rehabilitation, however, it must keep in mind that there are only 8 cases with denture rehabilitation, more high quality studies are needed to clarify this issue.

Interestingly, the patients with malignant tumors underwent radiation therapy in the three months following the surgery. The complications associated with adjuvant radiotherapy, including dry mouth, muscle fibrosis, and temporomandibular disorder [20], could have all had negative effects on masticatory performance [21]. On the other hand, the patients with benign tumors did not have to undergo this 3-month session of radiotherapy, additionally, the different resection extents between malignant and benign diseases could have also had an effect on the masticatory performance, as more essential muscles were excised in malignant patients.

In theory, fixed dentures can provide better functional results than removable dentures, and removable dentures can provide better functional results than when no rehabilitation is performed, as confirmed by previous studies [8, 9, 17, 22]; however, our finding did not support this finding. The most possible reason was that only benign patients wear denture, Leung et al. [23] previously stated after cancer ablation, the patients were only satisfied with soft tissue reconstruction but not reconstructed mandible because the flap was on longer innervated.

Subjective experience was another important aspect of assessing the functional results after OFF flap reconstructions. Zavala et al. [10] found the mean chew domain score to be 85.3 ± 22.7 in 29 patients who underwent OFF flap reconstructions for central defects; although most of the patient in this study received dental implant rehabilitation, the chew domain score was still less than that in our study at 12 months postoperatively. A type C defect was more likely to cause significant dysfunction than other types. Yang et al. [24] analyzed the quality of life in 34 patients who underwent an OFF flap for mandibular reconstruction, and they reported the chew domain score to be just 33.1 ± 16.1, which was lower than that in our study. But this study did not provide the preoperative chew domain data, and it also assessed the chew function at a certain time point. This study might be the first to dynamically analyze the subjective chew function. Although there was a transient reduction, all the subjective chew functions could return to preoperative levels, and this recovery was not related to denture rehabilitation. This finding, combined with other reports [10, 24, 25], suggests the OFF flap is an ideal and predictable treatment modality for mandible reconstruction.

Limitation in current study must be acknowledged, firstly, our sample size was not large enough, it might decreased our statistic power; second, we neglected the significance of the number of remaining teeth, it might affected our outcome, in our future work, we would perform another research to clarify this issue.

In summary, the masticatory performance and subjective chew function was significantly affected after OFF flap reconstruction in short term, but both could recover to preoperative levels within 9–12 months. Therefore, an OFF flap is suggested for mandible reconstruction for achieving satisfactory function results.

## Data Availability

All data generated or analyzed during this study are included in this published article. And the primary data could be achieved from the corresponding author.
